# Coxsackievirus A10 blocks autophagosome-lysosome fusion to promote viral nonlytic spread and inflammatory cytokine release

**DOI:** 10.1128/spectrum.00830-25

**Published:** 2025-10-30

**Authors:** Ruibin Wang, Hui Li, Lin Zhu, Yuping Zhang, Rongxia Zuo, Chen Liu, Jie Song, Yajie Hu

**Affiliations:** 1 Department of Pulmonary and Critical Care Medicine, The First People’s Hospital of Yunnan Province, Kunming, China; 2 Department of Pulmonary and Critical Care Medicine, The Affiliated Hospital of Kunming University of Science and Technology, Kunming, China; 3 National & Local Engineering Center for Infectious Biological Products, Institute of Medical Biology, Chinese Academy of Medical Science and Peking Union Medical College 165063 , Kunming, Yunnan, China; Wannan Medical College, Wuhu, Anhui, China

**Keywords:** coxsackievirus A10 (CV-A10), autophagic secretory pathway, nonlytic viral spread, inflammatory cytokine release, neuropathological complications

## Abstract

We aimed to demonstrate whether coxsackievirus A10 (CV-A10) perturbed the fusion between autophagosomes and lysosomes to cause viral nonlytic spread and inflammatory cytokine release at the blood-brain barrier (BBB), thereby contributing to neuropathological damage. CV-A10-induced autophagic secretory pathway at the cellular level was examined using western blot, immunofluorescence, and flow cytometry methods. Then, the mechanism of autophagic secretory pathway in the neuropathogenesis of CV-A10 infection was demonstrated by constructing a suckling mouse model and combining behavioral, pathological, and molecular biological methods. The expression levels of autophagic secretory pathway-related proteins, extracellular inflammatory cytokines, and viral particles were significantly increased during CV-A10 infection. Moreover, 3-methyladenine significantly impeded the levels of autophagic secretory pathway-related proteins and the release of inflammatory cytokines or viruses; GW4869 and chloroquine partially suppressed these changes, whereas bafilomycin A1 had no obvious effects on these changes, implying that the autophagic secretory pathway without endosome or lysosome involvement might represent a new mode of inflammatory cytokine release and virus spread from intracellular to extracellular environments. Finally, an *in vivo* suckling mouse model further found that treatment with GW4869 significantly relieved the clinical symptoms and mortality of CV-A16-infected mice and inhibited the spread of the virus and the release of inflammatory cytokines. Our findings provide the first evidence that CV-A10 might trigger the autophagic secretory pathway by limiting autophagosome-lysosome fusion to complete the transmembrane nonlytic transmission of the virus at the BBB and the secretion of inflammatory cytokines, ultimately accelerating neuropathological damage.

**IMPORTANCE:**

This study provided the first evidence that coxsackievirus A10 (CV-A10) might use the autophagic secretory pathway for nonlytic intercellular release to complete transmembrane transmission at the blood-brain barrier and inflammatory cytokine secretion to accelerate the formation of neuroinflammation in infected hosts, which not only gave us a new understanding of the neuropathogenesis caused by CV-A10 but also offered a promising target to develop CV-A10 antiviral drugs.

## INTRODUCTION

Hand, foot, and mouth disease (HFMD) is a common infectious disease that mainly affects children younger than 5 years of age and is usually caused by a group of nonpolio enteroviruses, mainly including enterovirus 71 (EV-A71), coxsackievirus A16 (CV-A16), CV-A10, and CV-A6 (1). The main manifestations are flu-like symptoms, vesicular exanthema of the hands, feet, mouth, and occasionally the buttocks, poor appetite, vomiting, and diarrhea (2). A small number of patients may rapidly develop severe complications, including neurological, cardiovascular, and respiratory problems, which can lead to significant morbidity and even death (3, 4). Recently, inactivated monovalent EV-A71 whole-virion vaccines were developed and approved for marketing in China (5). However, animal studies and human clinical trials have shown that while these vaccines can protect against EV-A71, they are not applicable for preventing other enterovirus-associated HFMD (5, 6). Thus, developing safe and potent multivalent inactivated vaccines is considered a promising way to curb HFMD. In recent years, on the basis of epidemiological studies, CV-A10 has been associated with increased occurrences of sporadic HFMD cases and outbreak events globally (7). CV-A10, a nonenveloped icosahedral virus with a diameter of approximately 24 nm–30 nm, is thought to cause more serious disease than previously assumed (8). Accumulating evidence has demonstrated that CV-A10 infection can lead to more severe clinical manifestations, such as onychomadesis, herpangina, hyperCKemia, encephalitis, acute flaccid paralysis, and neurorespiratory syndrome, and a higher number of fatalities (9). Moreover, since its genome is more variable, the coinfection of CV-A10 with other enteroviruses increases the chance of genetic recombination and the complexity of the disease, which has attracted further interest from the research community worldwide (10). Hence, understanding the pathogenic characteristics of CV-A10 and determining the key points of its pathogenesis, especially the lethal neuropathic mechanism, can not only provide valuable information for the development of multivalent vaccines but also provide a new biotarget for the early diagnosis and treatment of CV-A10-induced neuropathy.

Autophagy is an evolutionarily conserved cellular process that mediates the clearance of cellular components, such as lipids, aggregated proteins, and damaged organelles, which are recycled to maintain cellular homeostasis, and is classically viewed as a degradation function (11). In addition to the autophagic degradative pathway, there is also a nondegradative autophagic machinery that mainly regulates unconventional secretory processes known as the autophagic secretory pathway (12). Recent investigations have revealed that the process of the autophagic degradative pathway involves the use of formed organelles (such as autophagosomes or endosomes) to deliver cellular material to lysosomes for degradation, but the process of the autophagic secretory pathway can change these organelle destinations from fusion with lysosomes to fusion with the plasma membrane for the expulsion of cellular cargo (12, 13). A growing body of research has also revealed that the autophagic secretory pathway mediates a plethora of factors ranging from cytokines to granule contents and even viral particles (14). One of the earliest examples of the autophagic secretory pathway was the secretion of interleukin-1β (IL-1β), which was demonstrated to be sequestered by a microtubule-associated protein 1 light chain 3B (LC3B)-positive carrier that then fused with the plasma membrane to release it for the development of inflammation (15, 16). Additionally, the packaging of viral ribonucleic acid (RNA) within autophagosomes or endosomes, which directly fuse with the plasma membrane to transfer viral RNA to other cells via the autophagic secretory pathway, enhances nonlytic viral spread (13, 17, 18). Therefore, the autophagic secretory pathway is now considered an additional mode of cell-to-cell communication and a mode of immune surveillance for pathogens (13, 18). There is evidence that enteroviruses can utilize the autophagic secretory pathway to facilitate their transmission (19); this includes viruses poliovirus (20), coxsackievirus B (21), and Enterovirus D68 (22). However, the role of the autophagic secretory pathway in CV-A10 infection has not been elucidated.

In this study, we demonstrated through *in vitro* cell experiments that CV-A10 induced incomplete autophagy; that is, CV-A10 induced autophagosome formation but inhibited the fusion of autophagosomes with lysosomes. The subsequent assessment of molecules related to the autophagic secretory pathway revealed that the expression of these molecules was obviously elevated during CV-A10 infection and that the levels of extracellular inflammatory cytokines and viral particles were also significantly increased. We then used multiple inhibitors of the autophagy pathway to identify the process of incomplete autophagy. Inhibitors of the autophagic secretory pathway directly interfered with the levels of extracellular inflammatory cytokines and viral particles. Finally, we constructed an *in vivo* suckling mouse model to further reveal that CV-A10 promoted the transmission of the virus and the release of inflammatory cytokines through the autophagic secretory pathway, leading to neuropathogenesis.

## MATERIALS AND METHODS

### Cell culture and viral infection

Human umbilical vein endothelial cells (HUVECs; ATCC, USA), a human glioma cell line (U-87 MG cells; Procell, China), and African green monkey kidney cells (Vero cells; ATCC, USA) were maintained in Dulbecco’s modified Eagle’s medium (DMEM; Corning, USA) supplemented with 10% fetal bovine serum (FBS; Gibco, USA) and 100 µg/mL penicillin-streptomycin solution (Biosharp, China) at 37°C in a humidified 5% CO_2_ incubator. The next day, the culture medium was replaced with fresh complete DMEM. The cells were passaged every 3 days with 0.25% trypsin.

For virus infection, HUVECs were seeded in six-well plates at a concentration of 5 × 10^5^ cells/mL. After 24 h, CV-A10 (subgenotype C, GenBank no. MN557275), which was isolated during an epidemic in Xiangyang, China, in 2017, was inoculated into cells at a multiplicity of infection of 0.1 for 2 h at 37°C. The viral inoculum was then removed, and the wells were washed twice with sterile phosphate-buffered saline (PBS) and replenished with DMEM containing 2% FBS. The cells and supernatants were harvested at the indicated time points. In addition, we constructed an *in vitro* model of the blood-brain barrier (BBB) with Transwell chambers. U-87 MG cells were plated at the bottom of the 24-well plate, while HUVECs infected with CV-A10 were plated in the Transwell chambers and then placed into the 24-well plate.

For the cell treatments, rapamycin, ammonium chloride (NH_4_Cl), 3-methyladenine (3-MA), GW4869, chloroquine (CQ), and bafilomycin A1 were used in this study. Rapamycin, a potent and specific mammalian target of rapamycin inhibitor, is a common autophagy activator; NH_4_Cl is a lysosomotropic agent that increases the intralysosomal pH; 3-MA, an inhibitor of phosphatidylinositol-3-hydrokinase, is widely used as an inhibitor of autophagy; GW4869, a noncompetitive neutral sphingomyelinase inhibitor, is usually used as an inhibitor of exosome synthesis/release and has been reported to prevent the fusion of secretory autophagosomes with cell membranes (23); in this study, we regarded GW4869 as an inhibitor of the autophagic secretory pathway, and CQ as an inhibitor of autophagy by increasing the pH of acidic organelles (e.g., lysosomes and endosomes) and thus preventing the binding of autophagosomes and acidic organelles. Bafilomycin A1 is a specific, reversible vacuolar H⁺-ATPase inhibitor that blocks the fusion of autophagosomes and lysosomes. Therefore, these agents activated or inhibited autophagy through different mechanisms at different stages of the autophagy process (Fig. 8).

### Quantitative real-time polymerase chain reaction (qRT-PCR)

To examine miR-1303 expression, qRT-PCR was carried out as described previously. In brief, miRNAs were first isolated from samples according to the instructions of the miRcute miRNA Isolation Kit (TIANGEN, China). Subsequently, qRT-PCR was performed in triplicate for each sample with SYBR Premix Ex Taq (TAKARA, Japan) on an ABI 7500 real-time PCR system (Applied Biosystems, USA). U6 was amplified as an internal control. The primer pair (forward: 5′-TTTAGAGACGGGGTCTTGCTCT-3′, reverse: 5′-CAGTGCGTGTCGTGGAGT-3′) of miR-1303 and the primer pair of U6 (forward: 5′-GCTTCGGCAGCACATATA CTAAAAT-3′, reverse: 5′-CGCTTCACGAATTTGCGTGTCAT-3′) were used in the current study.

To determine the viral load, total RNA was first extracted following the instructions of the TIANamp Virus RNA Kit (TIANGEN, China). Then, qRT-PCR was conducted on CV-A10 standards with known viral concentrations and on each sample using a Coxsackievirus A10 RNA detection kit (TIANLONG, China) on a GENTIER 96 instrument (TIANLONG, China). Eventually, the viral load of each sample was calculated from the standard curve generated from the CV-A10 standards.

### Determination of the 50% tissue culture infectious dose (TCID_50_)

Viral titers were determined by a TCID_50_ assay. Briefly, Vero cells were seeded into 96-well plates at 1 × 10^4^ cells per well. The next day, all the collected supernatants at different infection time points were serially diluted 10-fold and added to 96-well plates with 80% confluent cells. After incubation for 2 h at 37°C, the inoculum was removed, the cells were washed with PBS, and DMEM containing 2% FBS was added to each well. The cells were observed daily for cytopathic effects, and the TCID_50_ was calculated by the Reed-Muench method.

### Western blotting (WB) analysis

The samples (treated cells and tissues) were washed twice with cold PBS and incubated on ice with RIPA lysis buffer (Beyotime, China) containing a protease inhibitor cocktail (Beyotime, China). The extracted proteins were quantified with a BCA protein assay kit (Beyotime, China) and denatured in 5× sodium dodecyl sulfate-polyacrylamide gel electrophoresis (SDS-PAGE) loading buffer at 100°C in boiling water for 10 min. Twenty micrograms of total protein was loaded on 10% SDS-PAGE gels, transferred to polyvinylidene difluoride membranes (Millipore, USA), and further blocked with 5% nonfat dry milk (Servicebio, China) for 2 h at room temperature. The membranes were then incubated with primary antibodies (1:1,000 dilution) against viral capsid protein 1 (VP1; GeneTex, China), matrix metalloproteinase 9 (MMP9; Affinity, USA), claudin 5 (Affinity, USA), zonula occludens 1 (ZO-1; Affinity, USA), occludin (Affinity, USA), VE-cadherin (Affinity, USA), LC3 (Abcam, USA), sequestosome 1 (also called p62; CST, USA), lysosome-associated membrane protein 1 (LAMP-1; ABclonal, China), Golgi reassembly stacking protein 65 (GRASP65; ABclonal, China), Ras-related protein 8A (RAB8A; Abcam, USA), tumor susceptibility gene 101 (TSG101; Abcam, USA), Alix (Abcam, USA), vesicle trafficking protein SEC22 homolog B (SEC22B; Abcam, USA), tripartite motif containing 16 (TRIM-16; Abcam, USA), and glyceraldehyde-3-phosphate dehydrogenase (Abbinke, China) overnight at 4°C. Following three washes with 0.1% Tween 20/PBS, the membranes were further incubated with horseradish peroxidase (HRP)-conjugated secondary antibodies at room temperature for 1 h and detected with enhanced chemiluminescence (Biosharp, China).

### Immunofluorescence (IF) staining

The cells were seeded on coverslips in a 24-well plate. After different treatments, the cells were fixed in 4% paraformaldehyde for 30 min, permeabilized with 0.1% Triton X-100 (Solarbio, China) for 15 min, and blocked in 5% bovine serum albumin (BSA; Solarbio, China) for 30 min at room temperature. Then, the cells were incubated with primary antibodies (1:100 dilution), including those against glial fibrillary acidic protein (GFAP; ABclonal, China), VP1, claudin 5, ZO-1, occludin, VE-cadherin, LC3, LAMP-1, mannose-6-phosphate receptor (M6PR; Abcam, USA), and GRASP65, at 4°C overnight. Following three washes with PBS, the cells were further incubated with Alexa Fluor 488-conjugated goat-anti-mouse IgG and Alexa Fluor 594-conjugated goat-anti-rabbit IgG (1:300 dilution; CST, USA) at 37°C for 1 h. Finally, 4´,6-diamidino-2-phenylindole was used to visualize the nuclei. The fluorescence signals were observed under a confocal microscope (Leica, Germany).

### Plasmids and transfection

To assess autophagy flux, plasmids containing green fluorescent protein (GFP)-LC3 and green fluorescent protein-red fluorescent protein (RFP-GFP)-LC3 were kindly provided by Professor Song Jie from the Institute of Medical Biology, Chinese Academy of Medical Sciences. Plasmid transfection was performed with Lipofectamine 3000 (Invitrogen, USA) according to the manufacturer’s protocol as previously described (24).

### Isolation of extracellular vesicles

Extracellular vesicles were isolated for subsequent assessment of vesicles released by the autophagic secretory pathway and to determine whether they contained viral particles and inflammatory cytokines. In this study, we used the commercial Capture Extracellular Vesicle Isolation Kit (Takara, Japan), following the manufacturer’s instructions to obtain extracellular vesicles.

### Assessment of inflammatory cytokines by flow cytometry

Twenty inflammatory cytokines, including tumor necrosis factor α (TNF-α), IL-12, IL-4, IL-17, IL-8, interferon γ (IFN-γ), IL-10, IL-1β, IL-6, IL-2, IFN-α, and IL-5, were detected with a Bio-Plex cytokine assay (RAISECARE, China) in accordance with the manufacturer’s instructions as previously described (25).

### Cell counting kit-8 (CCK-8) assays

In the previous literature, it was demonstrated that GW4869, as an exosome release inhibitor (26) or multivesicular body (MVB) formation inhibitor (27), was used to investigate the autophagy-mediated secretory pathway. Based on previous references to the dosage of GW4869, we also conducted a CCK-8 experiment to evaluate the dosage and cytotoxicity of GW4869 in this study. The cells were collected, and the cytotoxicity of different GW4869 dosages was measured using the commercial CCK-8 kit (Beyotime, China) according to the reagent manual provided by the manufacturer as previously done (28).

### Animal experiments

Pregnant BALB/c mice obtained from the Experimental Animal Center of the Institute of Medical Biology, Chinese Academy of Medical Sciences, were randomly divided into the following four groups (*n* = 13): the PBS group, GW4869 group, CV-A10 group, and GW4869+CV-A10 group. Follow-up experiments were carried out in 1-day-old neonatal mice. The control group and GW4869 group were given intraperitoneal injections of 100 µL of PBS and GW4869, respectively. We used 1-day-old suckling mice for infection experiments by intraperitoneal injection of 100 µL of diluted virus (10^6^ PFU), whereas in the GW4869+CV-A10 group, GW4869 was administered before intraperitoneal injection of the virus into the suckling mice. Mouse survival and clinical symptoms were monitored daily for 5 days. At 5 days post-infection, the mice from each group were sacrificed, and the viral copy numbers and locations of CV-A10 in the brain and lungs were measured by qRT-PCR and immunohistochemical (IHC) staining, respectively. The pathological changes in the brain and lung tissues were further observed via hematoxylin and eosin (H&E) staining. Supernatants from homogenized brain and lung tissue clarified by centrifugation were assayed for inflammatory cytokines using a microsphere immunoassay as described above.

### IHC staining for VP1 identification

Deparaffinized slides were subjected to antigen retrieval with citrate antigen retrieval solution (Servicebio, China) by microwaving for 10 min and were then treated with 5% BSA in PBS for 30 min at room temperature. After incubation with a CV-A10 VP1 protein monoclonal antibody (1:100 dilution) overnight at 4°C, the slides were incubated with an HRP-conjugated secondary antibody (1:800 dilution) for 1 h at room temperature, followed by staining with diaminobenzidine (Servicebio, China) for 15 min at room temperature. The stained slides were finally sealed with a neutral resin and photographed using a digital pathology slide scanner (KFBIO, China).

### Histological examination

Deparaffinized slides were subjected to H&E staining for histological examination. After sequential staining with hematoxylin for 5 min and eosin for 2 min at 25°C, 5-µm-thick sections were sealed with a neutral resin, and visual fields were randomly selected under a digital pathology slide scanner (KFBIO, China).

### Data analysis

All the data were obtained from three independent experiments for quantitative analysis and are expressed as the means ± standard errors of the means. Significance values were calculated by applying Student’s *t*-test between two groups or one-way ANOVA with Tukey’s multiple-comparisons test among multiple groups. Survival curves for different variable values were depicted according to the Kaplan-Meier method and compared across the levels of variables with the log-rank test. Complete linkage hierarchical clustering of the Euclidean distances between groups and the Euclidean distances between 12 cytokines was performed. The heatmap was generated with the R package pheatmap. *P*-values less than 0.05 were considered statistically significant.

## RESULTS

### CV-A10 traversed Transwell chambers containing HUVECs and entered U-87 MG cells

In an *in vitro* model of the BBB, the viral load, virus titer, and VP1 expression consistently increased in HUVECs over time (Fig. S1A), and these three indicators also gradually increased in the U-87 MG cells in the lower Transwell compartment (Fig. S1B), suggesting that CV-A10 might enter U-87 MG cells by traversing the upper Transwell chamber containing the HUVECs. Moreover, IF staining further showed that CV-A10 could infect U-87 MG cells (Fig. S1C). Meanwhile, compared with the uninfected U-87 MG cells (Fig. S1C, magenta arrow), the expression of GFAP in U-87 MG cells infected with CV-A10 was significantly upregulated. Given the previous studies, which have shown that GFAP serves as a marker for the activation of astrocytes (29), this result hinted that astrocytes can be activated after CV-A10 infection (Fig. S1C, white arrow). Thus, the above results revealed that both HUVECs and U-87 MG cells were highly susceptible to CV-A10, and an *in vitro* BBB model also revealed that CV-A10 could directly cross the BBB.

### CV-A10 infection did not alter HUVEC junctional complexes

**Fig 1:**
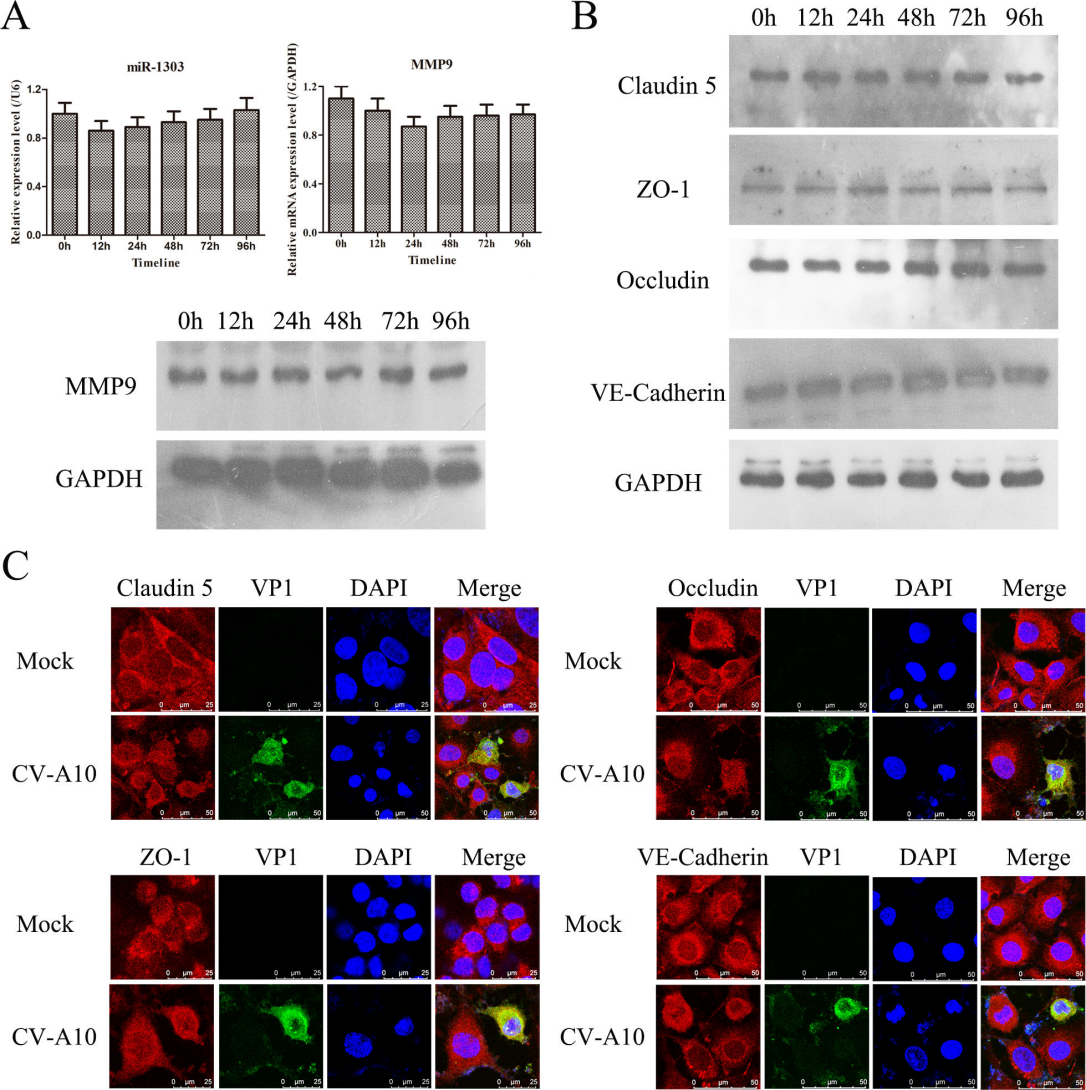
CV-A10 infection did not affect the expression of miR-1303, MMP9, or junctional complexes. (**A**) The expression levels of miR-1303 and MMP9 were examined in CV-A10-infected HUVECs via qRT-PCR and WB. (**B**) The expression of junctional complexes was examined in HUVECs via WB. (**C**) CV-A10-infected HUVECs were fixed and visualized by confocal microscopy after staining for VP1 and junctional complexes.

On the basis of the above results, we first hypothesized that CV-A10 altered HUVEC permeability in a manner similar to the enhancement of HUVEC permeability caused by CV-A16 infection reported in our previous study (30). Therefore, we investigated the BBB destruction pattern induced by CV-A10 in accordance with our previous study. The results revealed that the expression levels of miR-1303 and MMP9 were not significantly altered (Fig. 1A). Moreover, there were no significant changes in the protein levels (Fig. 1B) or location (Fig. 1C) of junctional complexes, namely claudin 5, occludin, ZO-1, and VE-cadherin. Hence, these findings implied that CV-A10 might cross the BBB in a different way than CV-A16 did.

### CV-A10 infection induced incomplete autophagy

**Fig 2:**
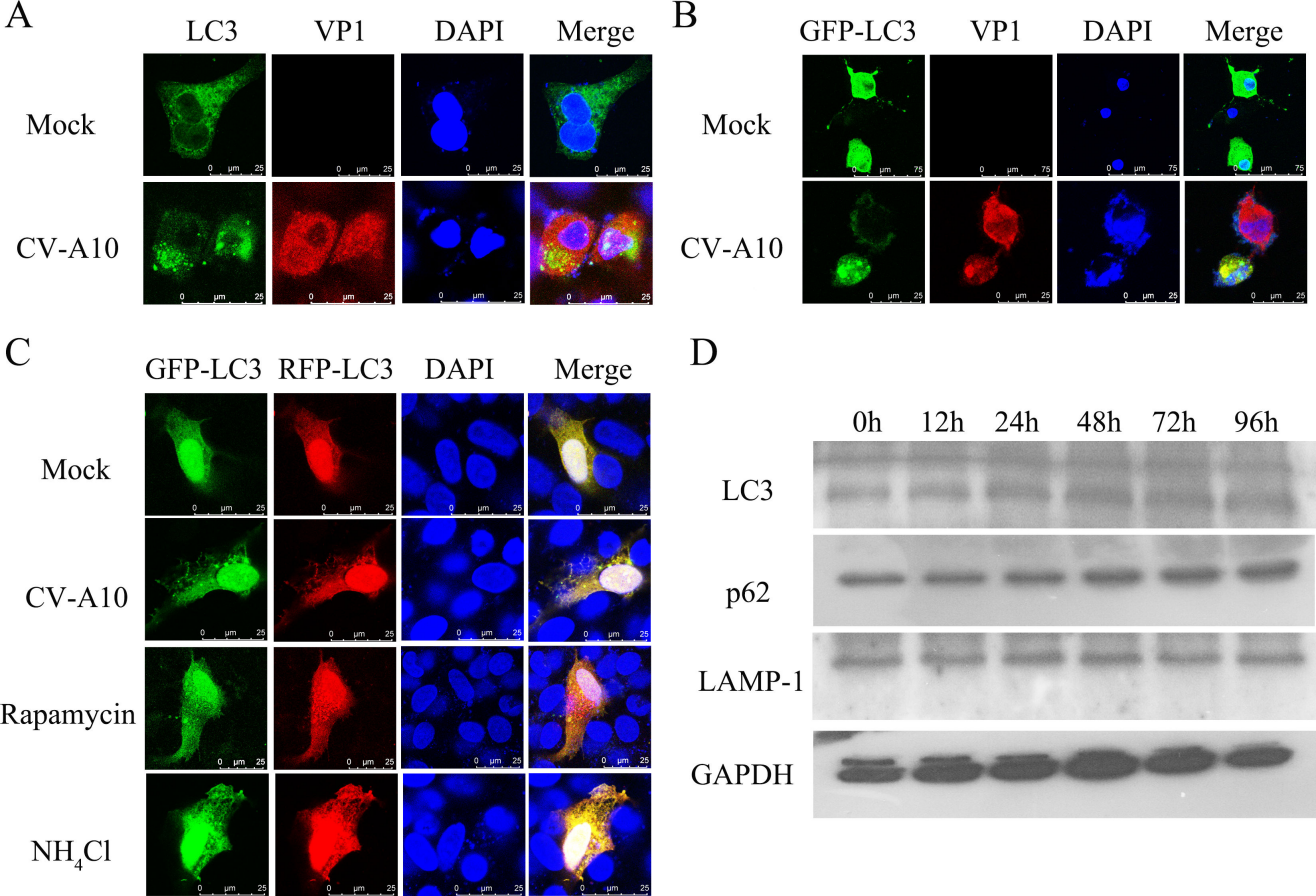
CV-A10 induced incomplete autophagy. (**A**) VP1 colocalized with endogenous LC3. (**B**) VP1 colocalized with exogenous LC3. (**C**) Cells were transfected with the RFP-GFP-LC3 plasmid. The yellow or red puncta indicate autophagosomes or autolysosomes, respectively. (**D**) WB analysis of LC3, p62, and LAMP-1 in CV-A10-infected HUVECs.

Endogenous and exogenous LC3 exhibited a diffuse distribution in the control group, whereas it presented a cytoplasmic punctate distribution, indicating the accumulation of autophagosomes in the CV-A10 group (Fig. 2A and B). To further explore whether CV-A10 induces complete autophagic flux, a vector encoding a GFP-RFP-LC3 reporter was transfected into cells. After transfection with the LC3 dual-fluorescence reporter plasmid, different results can be manifested through the display of different colors of fluorescence (31). When GFP-RFP-LC3-expressing cells undergo complete autophagy, in which autophagosomes fuse with lysosomes into autolysosomes, such as in the rapamycin group, only red puncta are present in the cells because the GFP signal is quenched in acidic lysosomes. However, when GFP-RFP-LC3-expressing cells undergo incomplete autophagy, such as in the NH_4_Cl group, the GFP signal does not enter the lysosome, and the red puncta perfectly overlap with the green puncta to form yellow puncta in cells, which represent autophagosomes. As shown in Fig. 2C, yellow puncta were observed in CV-A10-infected cells, which was in line with the findings in the NH_4_Cl group, suggesting that autophagosomes were successfully formed, whereas autolysosomes failed to form in CV-A10-infected cells. Furthermore, consistent with the results of IF staining, the levels of both LC3II and p62 continuously increased in CV-A10-infected HUVECs over time (Fig. 2D). Moreover, the level of LAMP-1, a lysosomal marker, did not markedly change (Fig. 2D). Therefore, these data indicated that CV-A10 might induce incomplete autophagy by blocking autophagosome fusion with lysosomes.

### CV-A10 infection triggered the autophagic secretory pathway

**Fig 3:**
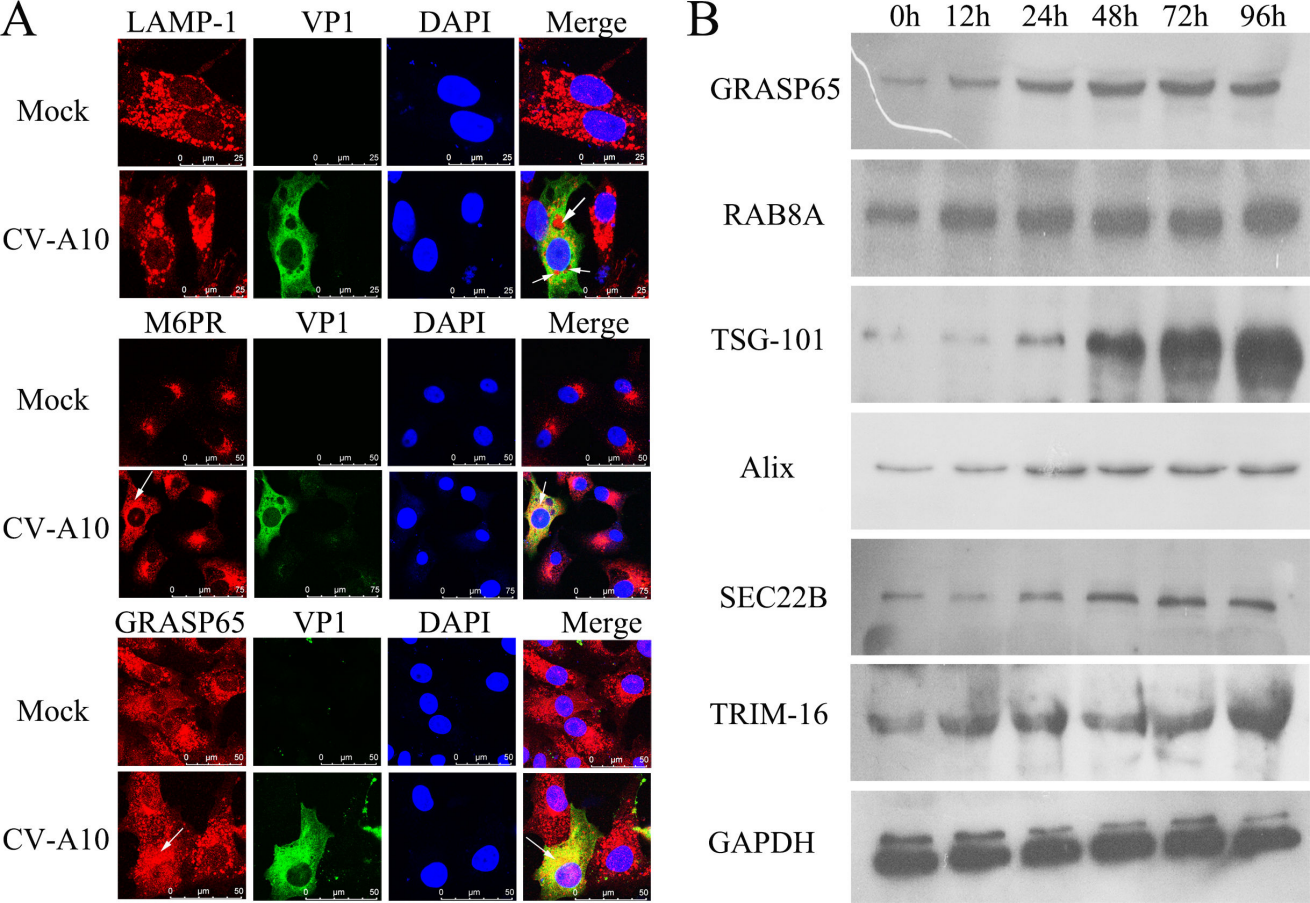
CV-A10 activated the autophagic secretory pathway. (**A**) Representative images of LAMP-1, M6PR, GRASP65, and VP1 staining in HUVECs. (**B**) Cell lysates were collected and subjected to a WB assay with specific antibodies against autophagic secretory pathway-related proteins.

To further clarify the abovementioned results, we directly used the IF staining technique to visualize the colocalization of the virus with lysosomes, endosomes, or secretory autophagosomes after CV-A10 infection. The markers of lysosomes, endosomes, and secretory autophagosomes used were LAMP-1 (32), M6PR (33), and GRASP65 (34), respectively. CV-A10 did not colocalize with LAMP-1 at all but did colocalize with M6PR and GRASP65 (Fig. 3A). The autophagic secretory pathway is a newly discovered nondegradative process of autophagy that occurs through the formation of autophagosomes that do not fuse with lysosomes (12, 34). These results suggested that the incomplete autophagy induced by CV-A10 might involve an autophagic secretory pathway. Next, according to the properties of the autophagic secretory pathway, we further examined its associated proteins, including GRASP65, RAB8A, TSG101, Alix, SEC22B, and TRIM-16, which were markedly increased. These proteins play important roles in the autophagic secretory pathway. For example, GRASP65 is considered to be an excellent marker for autophagic secretory pathway, as it is necessary for this process (35); RAB8A is a regulator of polarized sorting to the plasma membrane for autophagic secretory pathway (34); TSG101 functions as an autophagic secretory pathway receptor to capture the unconventionally secreted protein substrates into the amphisome (12); Alix is functionally required for efficient basal autophagy, especially in late endosome distribution and exosome biogenesis (36); and SEC22B, which interacts with TRIM-16, is needed to promote the secretory release of IL-1β, which is one of the earliest examples of a protein released extracellularly by autophagic secretory pathway (36, 37). Therefore, these data further suggested that CV-A10 might trigger the autophagic secretory pathway.

### Autophagic secretory pathway carried inflammatory cytokines and viral particles into the extracellular space during CV-A10 infection

**Fig 4:**
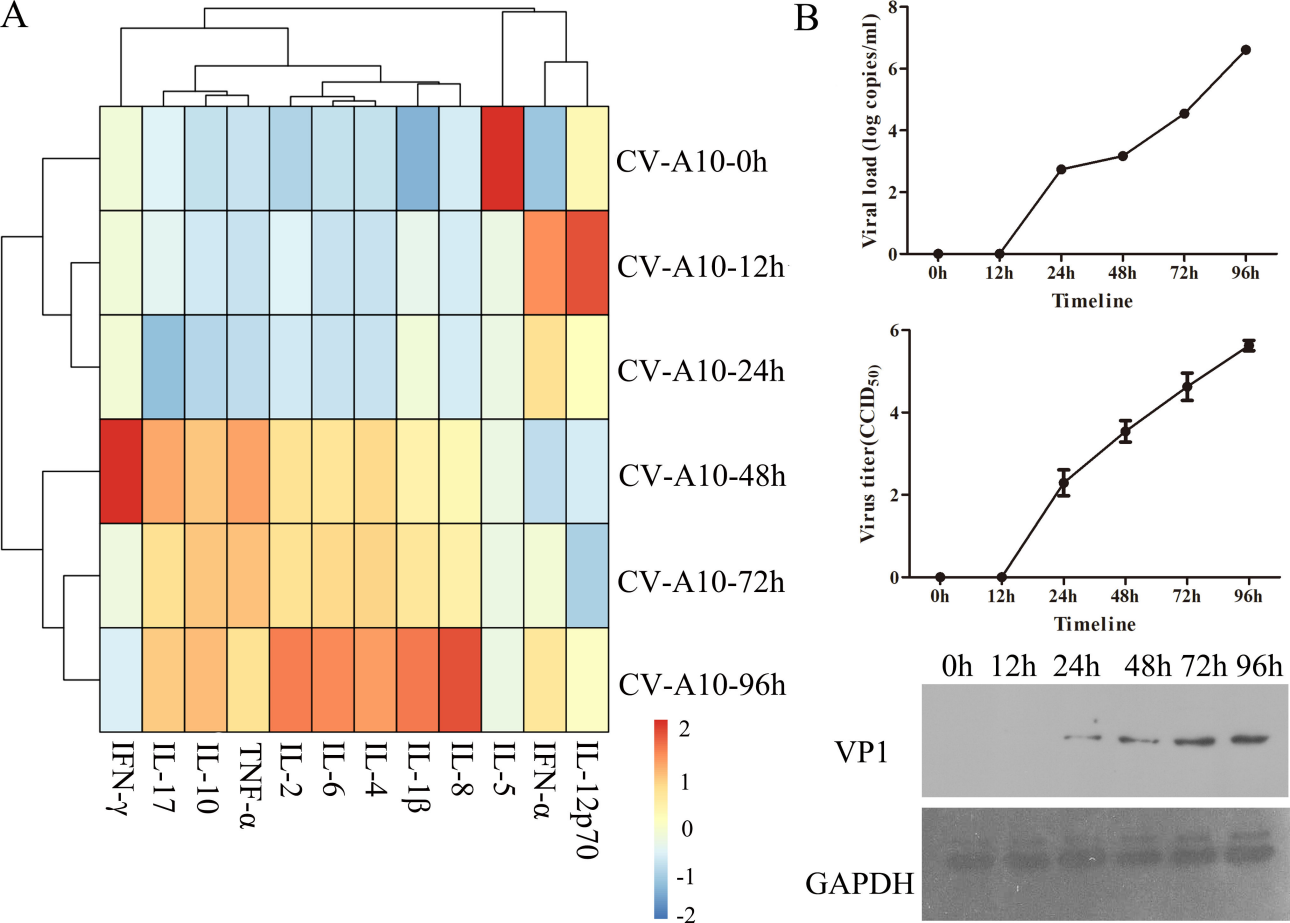
Inflammatory cytokines and viruses were detected in the extracellular vesicles of CV-A10-infected cells. (**A**) A heatmap presenting the results of inflammatory cytokines in extracellular vesicles by flow cytometry. (**B**) The proliferative dynamics of CV-A10 in extracellular vesicles were assessed by monitoring the viral load, virus titer, and VP1 expression via qRT-PCR, TCID_50_, and WB assays, respectively.

Recently, many studies have verified that the autophagic secretory pathway not only facilitates unconventional secretion of cytosolic cargo, especially IL-1β (16), but also mediates the nonlytic viral spread of some viruses (18). In this study, the supernatant of CV-A10-infected HUVECs was collected to assess the inflammatory cytokines and viral particles. The levels of 12 cytokines in each group were expressed as a heatmap (Fig. 4A). In the heatmap, each row represents a group. Each column represents a cytokine. The color of the tiles is used to represent the column Z-score of each cytokine. From the perspective of the color scale, the expressions of IL-8, IL-1β, IL-4, IL-6, IL-2, TNF-α, IL-10, and IL-17 showed a significant upward trend as the infection duration increased. Meanwhile, some inflammatory cytokines, such as IL-1β, IL-6, and IL-8, were obviously upregulated during CV-A10 infection with time (Table S1). Furthermore, the viral particles also began to be identified in the supernatant of CV-A10-infected HUVECs at 48 h after infection, and there was an increasing trend with time (Fig. 4B). Overall, our results revealed that the CV-A10-induced autophagic secretory pathway carried viral particles and inflammatory cytokines into the extracellular space, which might be one of the key factors accelerating viral transmission and the development of inflammation.

**Fig 5:**
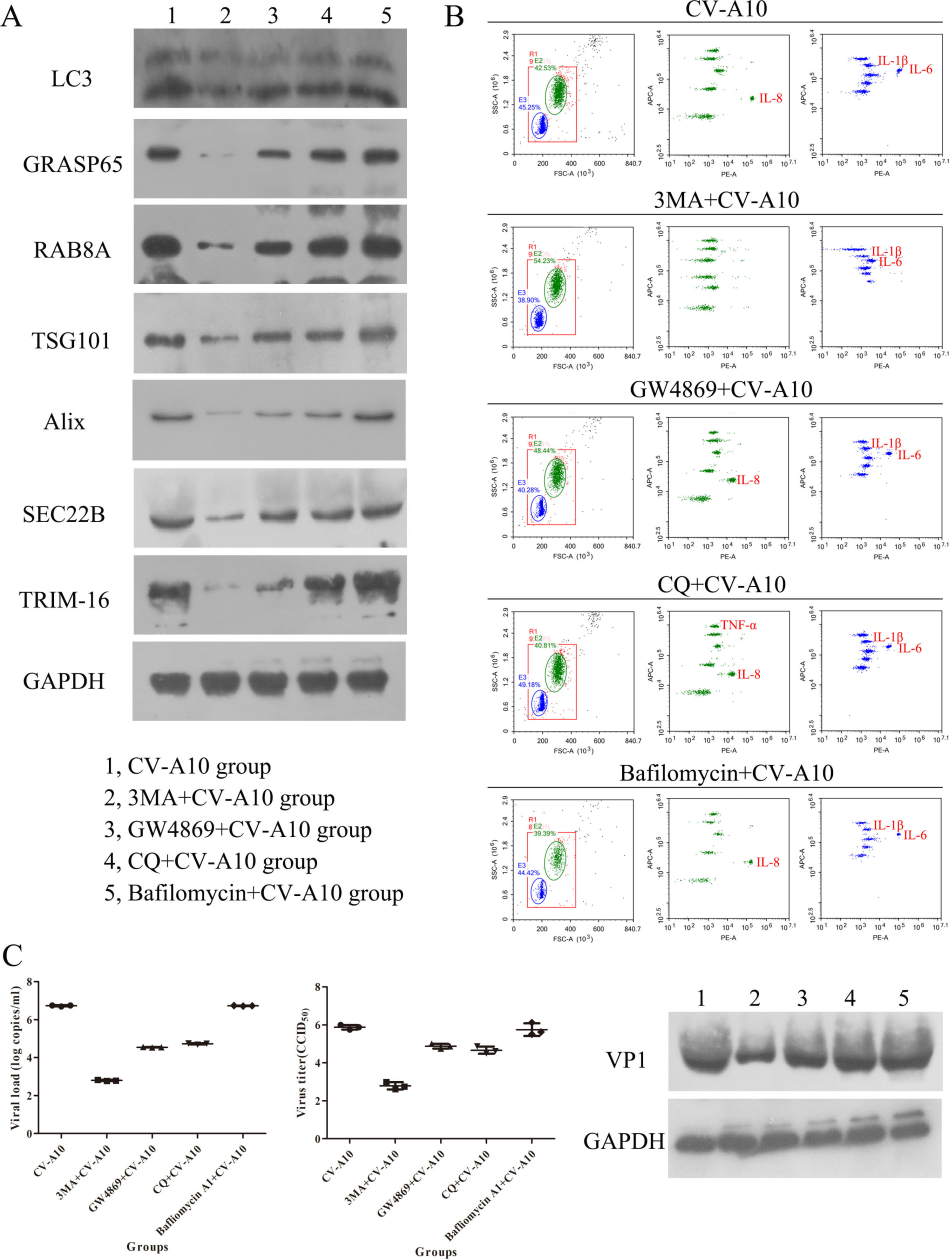
The autophagic secretory pathway was involved in the release of inflammatory cytokines and viruses during CV-A10 infection. (**A**) Protein expression levels of the autophagic secretory pathway were measured by WB in CV-A10-infected HUVECs subjected to different treatments. (**B**) A heatmap presenting the results of inflammatory cytokines in extracellular vesicles through flow cytometry in CV-A10-infected HUVECs subjected to different treatments. (**C**) Examination of extracellular viral RNA, virus titer, and VP1 protein from CV-A10-infected HUVECs subjected to different treatments by qRT-PCR, TCID_50_, and WB assays, respectively.

To further address the effect of the autophagic secretory pathway, we used 3-MA, GW4869, CQ, and bafilomycin A1. Compared with those in the CV-A10 group, the autophagic secretory pathway-associated proteins were sharply decreased in the 3-MA+CV-A10 and GW4869+CV-A10 groups and were partially decreased in the CQ+CV-A10 group; however, there were no significant differences between the CV-A10 group and the bafilomycin A1+CV-A10 group (Fig. 5A). In addition, the heatmap of flow cytometry revealed that, as compared to the CV-A10 group, some inflammatory cytokines, including IL-4, IL-17, IL-1β, IL-2, IL-8, and IL-6, were rapidly downregulated in the supernatants of CV-A10-infected cells treated with 3-MA, GW4869, and CQ, but these inflammatory cytokines had no significant effect under bafilomycin A1 treatment (Fig. 5B). Additionally, the detailed values of the 12 cytokines in each group clearly showed the differential changes, especially IL-1β, IL-6, and IL-8 (Table S2). Moreover, from the perspective of group clustering, the CV-A10 group can be clustered with the bafilomycin+CV-A10 group, while the GW4869+CV-A10 group can be clustered with the CQ+CV-A10 group, and at the same time, it showed significant differences from the 3-MA+CV-A10 group. Interestingly, we also noted that 3-MA, GW4869, and CQ all suppressed CV-A10 proliferation; 3-MA had the most dramatic effect on the inhibition of CV-A10 proliferation, whereas, compared with that in the CV-A10 group, CV-A10 proliferation was not affected in the bafilomycin A1+CV-A10 group (Fig. 5C). Consequently, our data revealed that the autophagic secretory pathway might play an important role in the release of inflammatory cytokines and viral particles from CV-A10-infected HUVECs.

### The autophagic secretory pathway influenced CV-A10 transmission and inflammation

**Fig 6:**
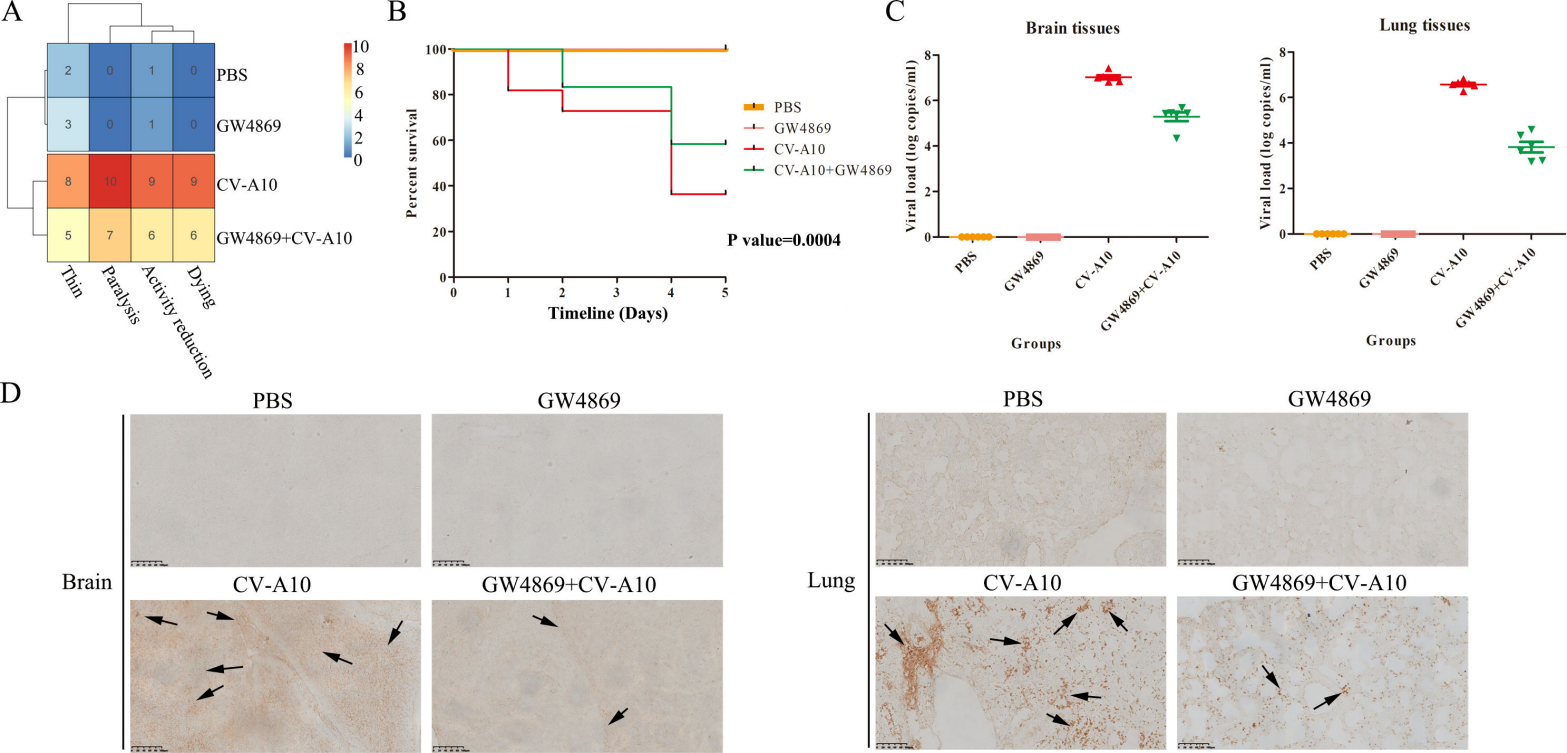
Suppression of the autophagic secretory pathway improved clinical manifestations, mortality rates, and infection rates. (**A**) The number of clinical symptoms in each group is expressed as a heatmap. In the heatmap, each row represents a group. Each column represents a clinical symptom. The color of the tiles is used to represent the number of clinical symptoms. (**B**) A 5 day survival curve was drawn. (**C**) Viral loads in the brain and lung tissues of the mice were tested via qRT-PCR. (**D**) IHC staining was conducted to determine the distribution of CV-A10, which is represented by black arrows.

**Fig 7:**
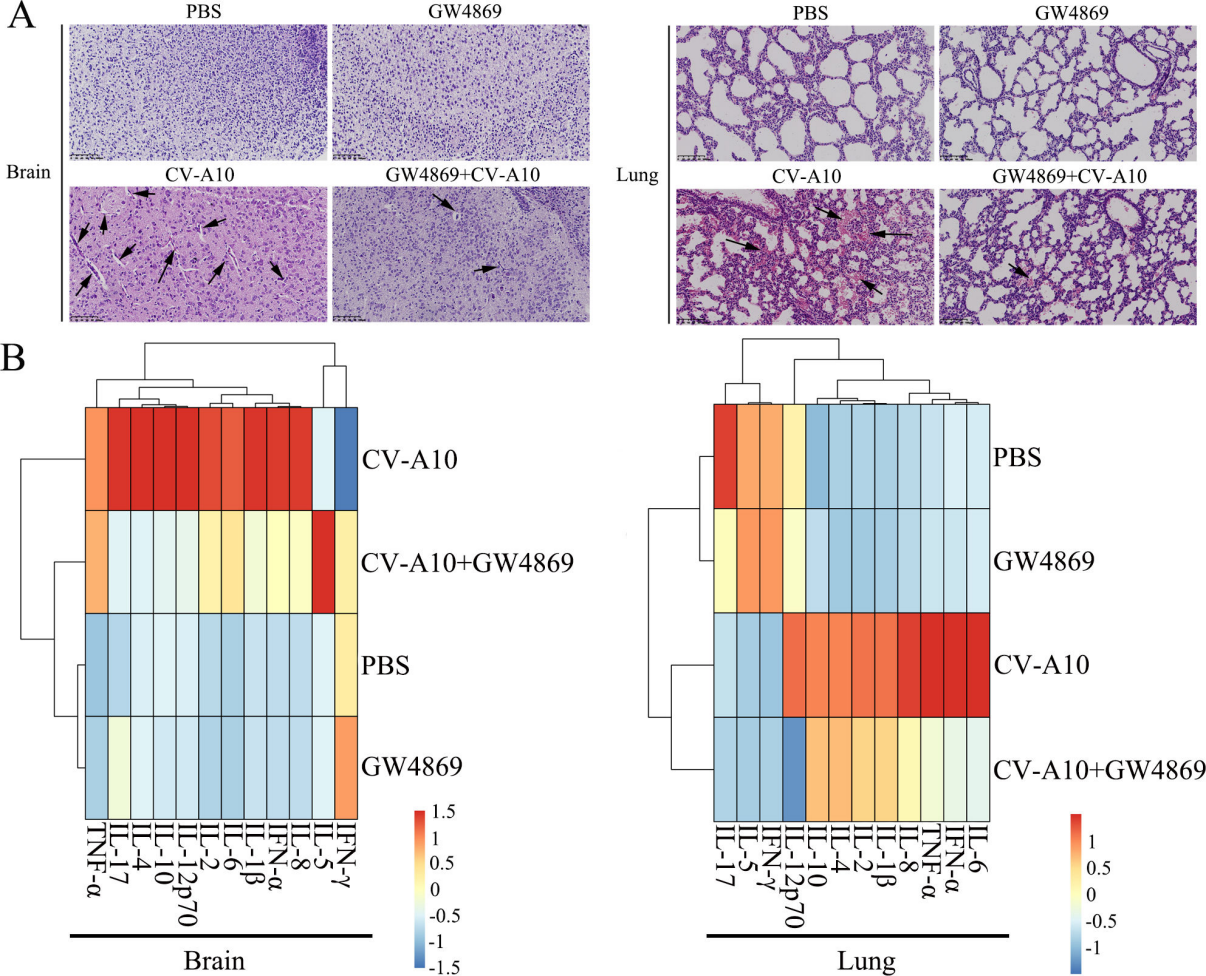
Inhibition of the autophagic secretory pathway ameliorated pathological injury and the release of inflammatory cytokines in the brain and lung tissues of CV-A10-infected mice. (**A**) The histopathological changes in the brain and lung tissues were analyzed by H&E staining and are indicated by black arrows. (**B**) A heatmap presenting the results of inflammatory cytokines in the brain and lung tissues of the mice via flow cytometry.

To further characterize whether the autophagic secretory pathway is involved in CV-A10 transmission and inflammation, we performed an animal experiment. The numbers in the heatmap represent the numbers of mice whose four different clinical symptoms changed across the different groups. The clinical symptoms of the mice in the CV-A10 group were clearly the most severe and were relieved after the administration of GW4869 (Fig. 6A). Moreover, the results also revealed that the administration of GW4869 significantly reduced the mortality of CV-A10-infected suckling mice (Fig. 6B). Additionally, both the viral load and VP1 expression in the brain and lung tissues of CV-A10-infected suckling mice were decreased when GW4869 was administered (Fig. 6C and D). Next, we monitored the changes in pathological and inflammatory cytokines in the brain and lung tissues of suckling mice. H&E staining revealed that CV-A10 infection led to hemorrhagic injuries, perivascular cuffing, and satellite phenomena in the brain and inflammatory cell infiltration, hyperemia, and structural destruction in the lung; however, these indicators markedly improved in the GW4869+CV-A10 group (Fig. 7A). Moreover, it is also seen that in brain tissues (Fig. 7B), from the perspective of group clustering, the PBS group and the GW4869 group were clustered together, while the CV-A10+GW4869 group did not cluster preferentially with the CV-A10 group. Instead, it clustered first with the PBS and GW4869 groups, indicating that there might be significant differences between the CV-A10+GW4869 group and the CV-A10 group. Meanwhile, it was also seen that the expressions of IL-8, IFN-α, IL-1β, IL-6, IL-2, IL-12p70, IL-10, IL-4, and IL-17 in the CV-A10+GW4869 group were remarkably lower than those in the CV-A10 group (Fig. 7B). However, in lung tissues (Fig. 7B), although the CV-A10 group clustered together with the GW4869+CV-A10 group, we can still observe that after the GW4869 treatment, some cytokines (i.e., IL-6, IFN-α, TNF-α, IL-8, IL-1β, IL-2, IL-4, IL-10, and IL-12p70) still showed significant downregulation. Meanwhile, the results of flow cytometry further revealed that GW4869 treatment resulted in a clear decrease in the release of inflammatory cytokines in the brain and lung tissues of the CV-A10-infected mice (Table S3). Taken together, these findings indicated that inhibition of the autophagic secretory pathway might restrict the transmission of CV-A10 and the formation of an inflammatory response induced by CV-A10 infection to a certain extent.

## DISCUSSION

CV-A10 has become the main causative agent of HFMD, causing mild, severe, and fatal cases in outbreaks or sporadic HFMD (8). A small proportion of severe or fatal CV-A10 cases are attributed primarily to the formation of neurological lesions or long-term neurological conditions, placing a huge burden on society and families (8, 9). Many studies have confirmed that disruption of the BBB is a vital property of neurotropic viral infection of the central nervous system (CNS) (38). For example, the West Nile virus disrupts the BBB in mice by degrading junctional complex proteins and increasing the number of multiple MMPs (39). Zika virus alters the expression of tight junction proteins to penetrate the brain parenchyma, resulting in BBB destruction (40). Rabies virus infection enhances BBB permeability by reducing tight junction protein expression and inducing the penetration of inflammatory cells into the CNS (41). Moreover, our previous study also demonstrated that CV-A16 crossed the BBB and then entered the CNS by downregulating miR-1303, which disrupted junctional complexes by directly regulating MMP9 (30). Thus, we wondered whether CV-A10, which is also an enterovirus, would enter the CNS in the same way as CV-A16. In this study, we first examined the expression levels of miR-1303, MMP9, and junctional complexes during CV-A10 infection. It was found that there were no significant changes in these molecules in CV-A10-infected HUVECs, suggesting that CV-A10 infection did not alter HUVEC junctional complexes through the miR-1303/MMP9 regulatory axis and did not further destroy BBB permeability. Based on this result, we hypothesized whether the CNS damage induced by CV-A10 infection could penetrate the BBB without destroying the BBB structure. Accumulating evidence demonstrated that the ways to regulate the invasion of neurotropic viruses into the CNS are not only by disrupting the integrity of the BBB but also through other means, such as a “Trojan horse” mechanism, etc. (42). Subsequently, we constructed an *in vitro* BBB model using the Transwell chamber in an attempt to verify our hypothesis. Our results clearly revealed that CV-A10 crossed from HUVECs in the upper Transwell chamber to U-87 MG cells in the lower Transwell chamber. These results led us to speculate that CV-A10 might cross the BBB in a novel mode that does not destroy the BBB structure and is also different from that used by CV-A16. According to previous research, the mechanisms by which neurotropic viruses cross the BBB to invade the CNS can be primarily classified into two major categories: the hematogenous route and the non-hematogenous route. The former mainly consists of the paracellular pathway (between cells), the transcellular pathway (through cells), or a “Trojan Horse” mechanism through diapedesis of infected immune cells, etc., whereas the latter mainly includes retrograde axonal transport and transynaptic trafficking (43).

It is generally believed in the field of research that the transmission of viruses between cells requires cell lysis (20). However, recent studies have revealed that nonlytic viral spread could be enhanced by autophagy components (20, 44). Actually, autophagy-dependent secretion has been found to affect the secretion of a plethora of factors ranging from cytokines to granule contents and even viral particles. For instance, it has been reported that viruses such as poliovirus (20), hepatitis C virus (45), and coxsackievirus B (21) have been found to utilize the autophagy-dependent secretion pathway as a novel means to support their release and spread of viral particles from host cells without the lysis method. Thus, we hypothesized in the current study whether the mode by which CV-A10 penetrates the BBB and invades the CNS utilizes this nonlytic autophagic secretory pathway for transmission.

It is well-known that autophagy is an evolutionarily conserved process that plays antiviral roles during viral invasion (46, 47). However, coevolution and coadaptation between viruses and autophagy have armed viruses with multiple strategies to subvert the autophagic machinery to escape host immunity and promote viral replication (47). The autophagic secretory pathway is a newly discovered way in which autophagosomes directly engulf and expel molecules with no leaders/signal peptides or intracellular pathogens to the external milieu instead of following conventional routes toward lysosomes (12). Recently, the alternative function of autophagy has been gaining more attention due to the fact that it contributes to the unconventional secretion of and viral particles (13, 48). The most special feature of autophagic secretory pathway-mediated viral transmission is that it does not lead to infection-related cell lysis and allows the virus to complete its intercellular spread. Based on the above research results, we incorporated the autophagic secretory pathway into the consideration of CV-A10’s transmembrane crossing of the BBB without destroying it. Previous studies have proven that the main difference between the secretory autophagy pathway and the classical degradation autophagy pathway lies in the fact that the autophagosomes formed in the degradative autophagy pathway directly fuse with the lysosomes, while the autophagosomes formed in the secretory autophagy pathway do not fuse with the lysosomes but directly fuse with the plasma membrane; thereby whether the autophagy flux is completed or not may be particularly important for distinguishing the secretory autophagy pathway from the degradative autophagy pathway (16). Autophagy flux refers to the integrity of the autophagy process and is used to measure the degree of autophagic degradation (49). If the binding process of the autophagosome and lysosome is obstructed, the autophagy flux is impaired and the material degradation process cannot be completed (50); meanwhile, it may also develop toward the secretory autophagy pathway. Accordingly, autophagy flux was first monitored. Our IF staining results revealed that CV-A10 infection induced puncta of endogenous and exogenous LC3, indicating the occurrence of autophagy. Then, we also observed an increase in the number of autophagosomes (GFP+ RFP+ puncta, representing no fusion events occurred between the autophagosomes and the lysosomes) compared with the number of autolysosomes (GFP-RFP+ puncta, representing fusion events occurred between the autophagosomes and the lysosomes) during the course of CV-A10 infection, suggesting that CV-A10 might induce incomplete autophagy. Moreover, the upregulated levels of expression of LC3II and p62 further supported the above inference. LC3 is a marker of autophagy, and its function is involved mainly in the formation of autophagosomes (51), whereas p62 is an important selective autophagy adaptor protein and is degraded during complete autophagy, but it is important to note that if p62 is not degraded, it indicates that autophagy activity is inhibited (52). Hence, this increase in the LC3II/I ratio represented the formation of autophagosomes, and the expression of the undegraded p62 signified that degradative autophagy was not fully induced. Meanwhile, LAMP-1, as a marker of lysosomes and also required for the fusion of lysosomes with phagosomes (53), did not show any significant changes during CV-A10 infection, which might imply that the lysosomes were not damaged. As mentioned above, if the autophagosome does not enter the lysosome to perform the degradation process, it may be redirected for secretion (12). Hence, we subsequently examined the related molecules of the autophagic secretory pathway. Firstly, the colocalizations of CV-A10 with the lysosome, endosome, and secretory autophagosome were evaluated. It was found that CV-A10 colocalized with M6PR (an endosome marker) and GRASP65 (a secretory autophagosome marker) but did not show any colocalization with LAMP-1, which pointed out that CV-A10 did not enter the lysosome for degradation, but instead entered into the secretory endosome or autophagosome. Then, the levels of key proteins involved in the autophagic secretory pathway were also measured. GRASP65 and RAB8A, which are necessary for autophagic secretory pathway, are involved in the regulation of cargo transfer to the Golgi and vectorial sorting to the plasma membrane, respectively (16). TSG-101 and Alix are markers of MVBs, exosomes, and extracellular vesicles (54, 55). TRIM-16 functions as a cargo receptor for autophagic secretory pathway through the binding of SEC22B to autophagosomes (48). It was discovered that all the above proteins were markedly increased during CV-A10 infection, which implied that the autophagic secretory pathway might be activated following CV-A10 infection.

In order to further analyze whether the autophagic secretory pathway induced by CV-A10 infection promotes the occurrence of inflammation and nonlytic spread of CV-A10, we examined the expression of inflammatory cytokines and the dynamics of viral proliferation in isolated extracellular vesicles, respectively. As mentioned earlier, the autophagic secretory pathway involves the process of secreting encapsulated substances into the extracellular fluid with the help of the autophagosome (16). Many inflammatory cytokines, such as IL-1β, HMGB1, and IL-18, complete their nonlytic export through the autophagic secretory pathway (13, 16, 27). Our data revealed that the levels of most inflammatory cytokines, such as IL-8, IL-1β, IL-4, IL-6, and IL-2, etc., persistently increased with time during CV-A10 infection. Normally, virus infection triggers a first wave of inflammatory cytokines for viral clearance, but excessive production of cytokines can in turn cause serious immunopathological injuries (56, 57); therefore, sustained inflammatory cytokine release may further exacerbate the pathological consequences of inflammation caused by CV-A10 infection. Moreover, the *in vitro* BBB model revealed that after CV-A10 infection of HUVECs in the upper Transwell chamber, inflammatory cytokines were released and directly acted on U-87 MG cells in the lower Transwell chamber, which also suggested that inflammatory cytokines stimulated by CV-A10 infection might cross the BBB and enter the CNS to activate neuroinflammation. Moreover, the viral load, virus titer, and VP1 expression, which were monitored at 24 h after CV-A10 infection, gradually increased with time, which implied that CV-A10 might be released from cells of the upper Transwell chamber in a nonlytic mode by packaging in extracellular vesicles to cells in the lower Transwell chamber. However, it is undeniable that our research results merely conducted relevant verification from multiple dimensions from an indirect perspective. It is well-known that using electron microscopy or immunogold labeling methods to directly visualize the CV-A10 virus particles encapsulated in the extracellular vesicles can directly confirm that CV-A10 is released through the autophagic secretory pathway. In this study, we adopted an indirect approach for the argumentation, which was as follows: we extracted the extracellular vesicles from the cell supernatant and then measured their viral copy number and the expression level of the viral VP1 protein. Indeed, electron microscopy or immunogold labeling methods are much more convincing than RNA extraction from extracellular vesicles to guide future studies. Nevertheless, due to the limitations of our laboratory conditions, we are currently unable to carry out this experimental operation.

**Fig 8:**
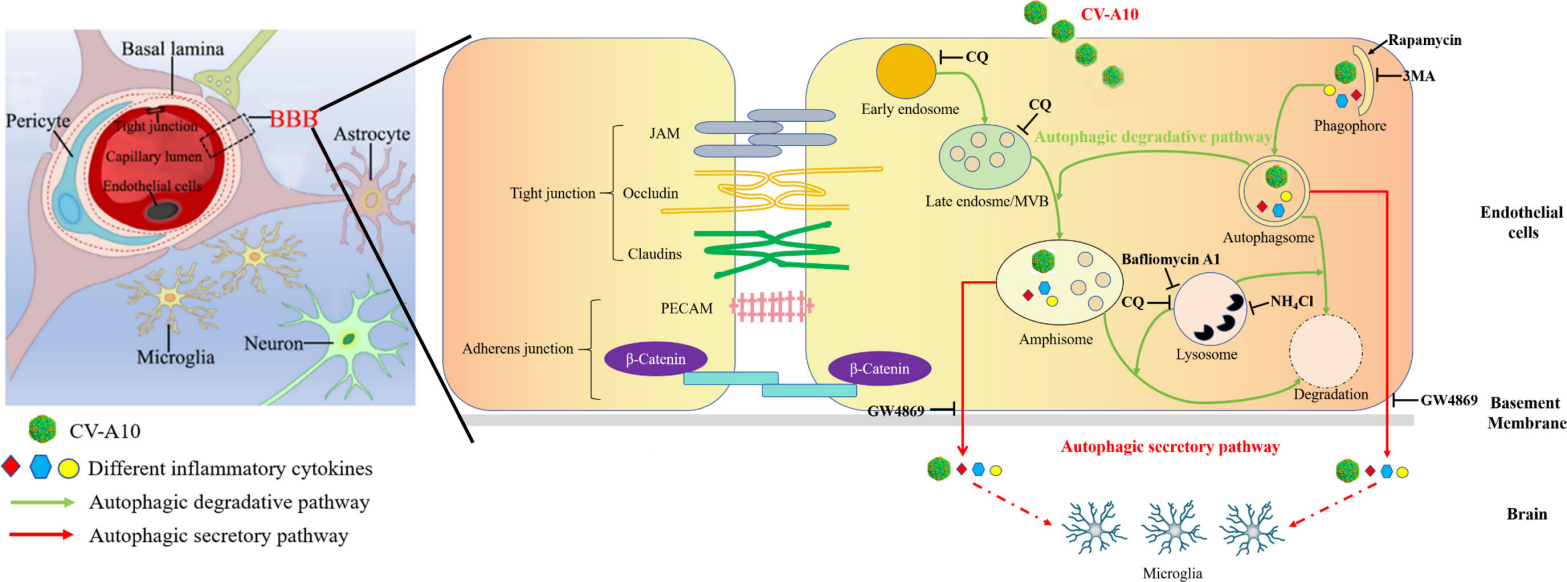
Model of the CV-A10-induced autophagic secretory pathway. On the left is a model diagram of the BBB, and on the right is a magnified BBB diagram. The focus of this diagram is the simulated progression of CV-A10 in endothelial cells after entry. The green arrow represents the autophagic degradative pathway, and the red arrow represents the autophagic secretory pathway. ┫ represents an inhibitor in the pathway, and → represents an activator in the pathway.

To further explore the key role of the autophagic secretory pathway, we subsequently used four different autophagy-related modulators (Fig. 8) to distinguish the effects of the autophagic secretory pathway on the release of inflammatory factors and viral particles after CV-A10 infection. Our results revealed that when autophagy was completely blocked through 3-MA treatment, the expressions of autophagic secretory pathway-related proteins, including LC3, GRASP65, RAB8A, TSG101, Alix, SEC22B, and TRIM-16, were notably decreased, but blockade of autophagosome and lysosome fusion through bafilomycin A1 treatment had no significant effect on the expressions of these proteins. In addition, when a secretory process-related inhibitor (i.e., GW4869) or acid organelle inhibitor (i.e., CQ) was administered, the expressions of these autophagic secretory pathway-related proteins were also suitably downregulated. These results first indirectly inform us that CV-A10 infection could induce the formation of early autophagy, but it could not promote the complete formation of degradative autophagy. Instead, it might facilitate the formation of the autophagic secretory pathway. Since we had initially determined that CV-A10 infection triggered the formation of the autophagic secretory pathway, we then immediately analyzed the inflammatory cytokines and viral particles present in the isolated extracellular vesicles under these treatments. The results revealed that in comparison to the CV-A10 group, the treatment with 3-MA, GW4869, or CQ all significantly reduced the inflammatory cytokines and virus particles in isolated extracellular vesicles after CV-A10 infection, whereas bafilomycin A1 treatment had no significant effect on these indicators. Therefore, our findings, to a certain extent, provided an indirect reflection that the CV-A10 infection process did not involve the pathway through which bafilomycin A1 works, but the treatment with 3-MA, GW4869, or CQ could affect the CV-A10 infection process. The schematic diagram in Fig. 8 clearly shows the autophagic degradative pathway and autophagic secretory pathway to fully understand the autophagic secretory pathway induced by CV-A10 infection. The autophagic degradative pathway begins with the formation of crescent membrane structures known as phagophores, which progressively surround cytoplasmic material and expand to form an enclosed double-membrane vesicle termed an autophagosome, which ultimately fuses with the lysosome for degradation (58, 59). However, unlike in the autophagic degradative pathway, the autophagosome does not fuse with the lysosome and instead directly fuses with the plasma membrane in the autophagic secretory pathway, which eventually results in the nonlytic extracellular release of cytoplasmic contents engulfed by the autophagosome (12, 60). In addition, autophagosomes can merge with late MVBs to produce amphisomes, which then further either fuse with lysosomes to degrade their contents or with the plasma membrane to release their contents (61). Taken together, our data hinted that the autophagic secretory pathway under 3-MA treatment, which plays an inhibitory role in the early stage of autophagy, was completely blocked, resulting in a marked reduction in the secretion of inflammatory cytokines and nonlytic release of viruses. Similarly, GW4869 mainly prevented the fusion of autophagosomes with the plasma membrane in the autophagic secretory pathway; that is, GW4869 inhibited the secretion process, and therefore, GW4869 treatment produced results similar to those of 3-MA treatment. Moreover, CQ primarily plays a role in inhibiting acidic organelles of the autophagy pathway, which means that CQ also partially inhibits the autophagic secretory pathway; thus, our results showed that CQ treatment also partially suppressed the secretion of inflammatory cytokines and the nonlytic release of viruses. However, bafilomycin A1 plays an inhibitory role in the late stage of autophagic degradation by repressing the fusion of autophagosomes and lysosomes, but since CV-A10 infection inhibited autophagy flux, bafilomycin A1 treatment did not affect the process of the autophagic secretory pathway induced by CV-A10 infection, and there were no obvious changes in the release of inflammatory cytokines and viral particles outside the cell. Altogether, to a certain extent, our study revealed that CV-A10 blocked autophagy flux by interfering with the fusion between autophagosomes and lysosomes and further induced autophagic secretion, which further promoted the production of inflammatory cytokines and CV-A10 destined for the extracellular space.

On the basis of the above data, the effects of the autophagic secretory pathway were further verified in animal experiments. The dosage of GW4869 was determined by further referring to relevant literature (26, 27) and the results of the CCK-8 experiment (Fig. S2). The administration of 10 µM GW4869 did not have a significant effect on cell viability. Upon GW4869 treatment, the clinical symptoms and death rate of CV-A10-infected suckling mice were obviously alleviated. Furthermore, growing evidence suggests that the most serious damage caused by CV-A10 infection is located in the CNS, and that neurogenic pulmonary edema originating from CNS damage is the main cause of death in CV-A10-infected children (8, 62). Therefore, our study focused on changes in the brains and lungs of these mice. The severe consequences of viral infection are clearly linked to viral load. Our data showed that the viral load in the brains and lungs of CV-A10-infected suckling mice treated with GW4869 was lower than that in the brains and lungs of CV-A10-infected suckling mice. Moreover, similar results were obtained from IHC staining, which suggested that CV-A10 infection might facilitate the activation of the autophagic secretory pathway for CV-A10 spread, but GW4869 treatment effectively reduced the invasion of CV-A10 into brain tissue and lung tissue. Meanwhile, it was also seen that GW4869 treatment could effectively alleviate hemorrhagic injuries, perivascular cuffing, and satellite phenomena in the brain, and inflammatory cell infiltration, hyperemia, and structural destruction in the lungs caused by CV-A10 infection, indicating that inhibition of the autophagic secretory pathway impeded the transmission of CV-A10 and also reduced pathological damage to the brains and lungs of suckling mice. Finally, previous studies reported that the host inflammatory response to CV-A10 infection is related to the brain and lung injuries induced by CV-A10 (38). Therefore, brain and lung tissues of suckling mice were ground to assess the expression of inflammatory cytokines. The results showed that CV-A10 infection accelerated the secretion of inflammatory cytokines, but GW4869 treatment directly diminished the levels of inflammatory cytokines, which further revealed that inflammatory cytokines might play an important role in CV-A10 infection through the autophagic secretory pathway.

In conclusion, our current study demonstrated that CV-A10 might trigger the autophagic secretory pathway by limiting autophagosome-lysosome fusion to complete the transmembrane nonlytic transmission of the virus at the BBB and the secretion of inflammatory cytokines, ultimately accelerating neuropathological complications, but not influencing the lysosomal dysfunction. Actually, increasing evidence has revealed that the autophagic secretory pathway is a strategic alternative to lysosomal degradation to export a range of cytosolic contents, including inflammatory cytokines and viruses (63). Thus, we first explored the autophagic secretory pathway triggered by CV-A10 infection at the cellular level, and then, by integrating the cellular level with the animal level, we further investigated that the autophagic secretory pathway triggered by CV-A10 infection promoted its nonlytic transmission and the release of inflammatory factors. Accordingly, the abovementioned results partially contribute to the understanding of the regulatory mechanism of CV-A10 spread and the release of inflammatory cytokines, which not only clarifies the mechanism by which CV-A10 crosses the BBB to evoke neuroinflammation but also may be useful for designing antiviral or anti-inflammatory drugs.

## Data Availability

All data generated or analyzed during this study are available from the corresponding author on reasonable request.

## SUPPLEMENTAL MATERIAL

Fig. S1 spectrum.00830-25-s0001.tif Susceptibility of HUVECs and U-87 MG cells to CV-A10.

Fig. S2 spectrum.00830-25-s0002.tif Results of the CCK8 experiment.

Supplemental Legends spectrum.00830-25-s0003.docx Legends for supplemental material.

Table S1 spectrum.00830-25-s0004.docx Identification of inflammatory cytokines at different points during CV-A10 infection.

Table S2 spectrum.00830-25-s0005.docx Identification of inflammatory cytokines in CV-A10-infected HUVECs with different treatments.

Table S3 spectrum.00830-25-s0006.docx Identification of inflammatory cytokines in brain and lung tissues of mice.

ASM does not own the copyrights to Supplemental Material that may be linked to, or accessed through, an article. The authors have granted ASM a non-exclusive, world-wide license to publish the Supplemental Material files. Please contact the corresponding author directly for reuse.
