# Supplementary figures and images for: Coxsackievirus A10 blocks autophagosome-lysosome fusion to promote viral nonlytic spread and inflammatory cytokine release
Source: Microbiol Spectr. 2025 Oct 30;13(12):e00830-25. doi: 10.1128/spectrum.00830-25 (PMC12671134; doi:10.1128/spectrum.00830-25)

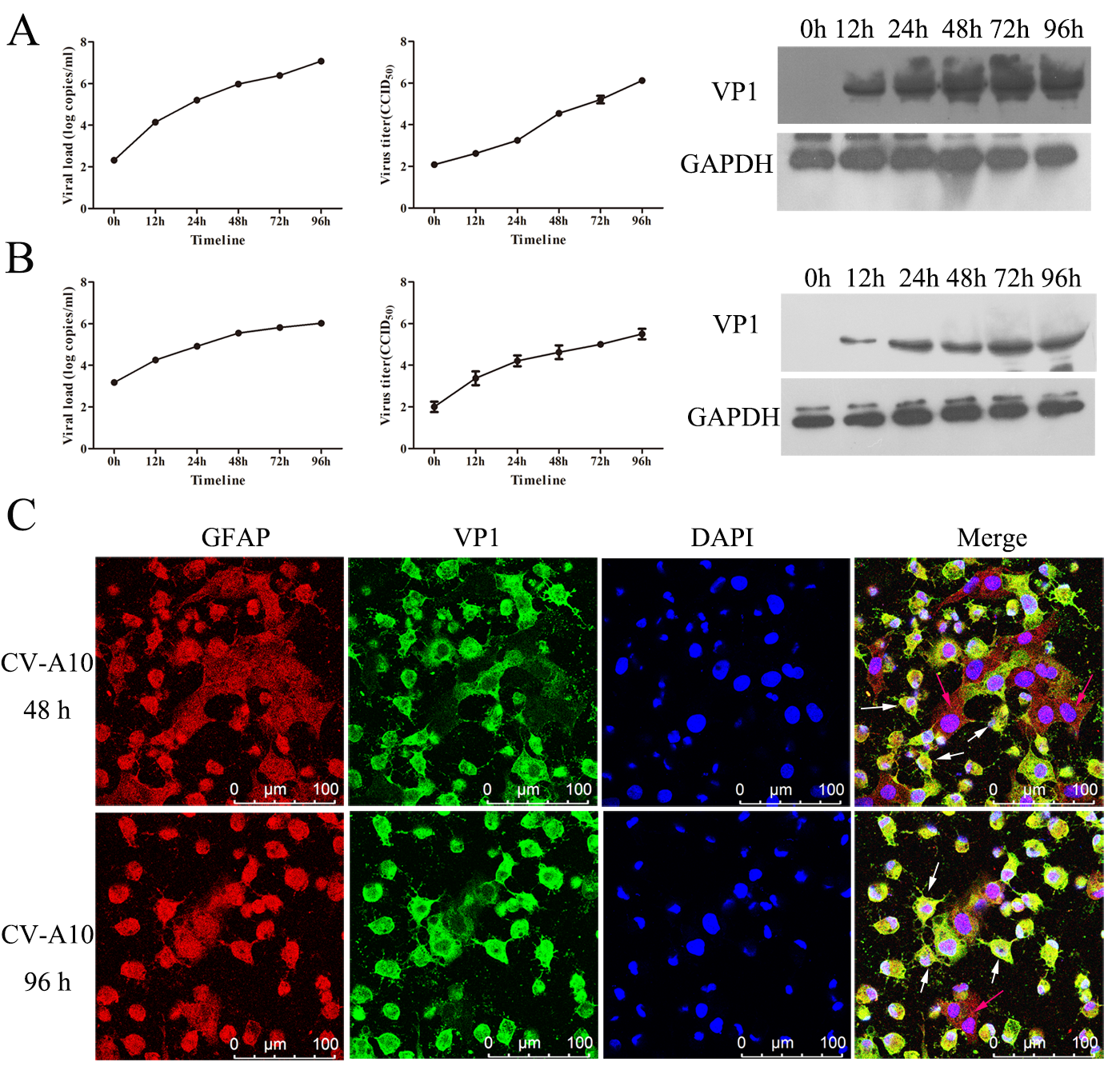

Supplement: Fig. S1 — Susceptibility of HUVECs and U-87 MG cells to CV-A10. [file spectrum.00830-25-s0001.tif]

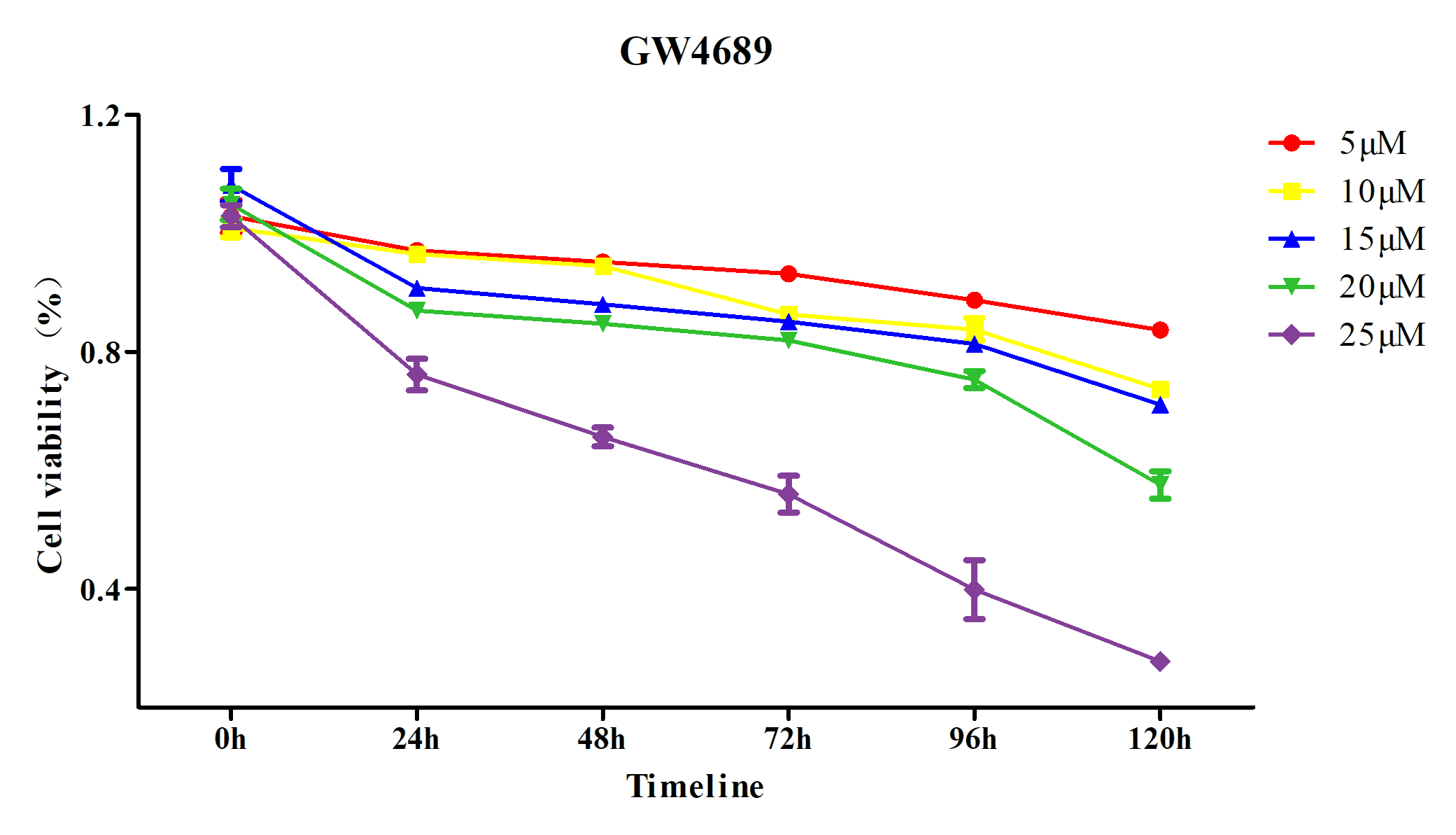

Supplement: Fig. S2 — Results of the CCK8 experiment. [file spectrum.00830-25-s0002.tif]
