# Supplementary material for: Coxsackievirus A10 blocks autophagosome-lysosome fusion to promote viral nonlytic spread and inflammatory cytokine release
Source: Microbiol Spectr. 2025 Oct 30;13(12):e00830-25. doi: 10.1128/spectrum.00830-25 (PMC12671134; doi:10.1128/spectrum.00830-25)
Supplement: Supplemental Legends — Legends for supplemental material. [file spectrum.00830-25-s0003.docx]

Supplementary Material:

Fig. S1 Susceptibility of HUVECs and U-87 MG cells to CV-A10. (A) Growth kinetics of CV-A10 in HUVECs were assessed. (B) Growth kinetics of CV-A10 in U-87 MG cells were evaluated. (C) IF staining was used to determine the infection rate of CV-A10. The magenta arrow indicates uninfected U-87 MG cells and the white arrow indicates CV-A10-infected U-87 MG cells.

Fig. S2 **The effects of GW4869 in cell viability via CCK test.**

Table S1. Identification of inflammatory cytokines at different points during CV-A10 infection.

**Table S2.** **Identification of inflammatory cytokines in CV-A10-infected HUVECs with different treatments.**

**Table S3. Identification of inflammatory cytokines in brain and lung tissues of mice.**
