# Supplementary material for: Coxsackievirus A10 blocks autophagosome-lysosome fusion to promote viral nonlytic spread and inflammatory cytokine release
Source: Microbiol Spectr. 2025 Oct 30;13(12):e00830-25. doi: 10.1128/spectrum.00830-25 (PMC12671134; doi:10.1128/spectrum.00830-25)
Supplement: Table S1 — Identification of inflammatory cytokines at different points during CV-A10 infection. [file spectrum.00830-25-s0004.docx]

**Table S1. Identification of inflammatory cytokines at different points during CV-A10 infection.**

| Groups | IL-5 | IFN-α | IL-2 | IL-6 | IL-1β | IL-10 | IFN-γ | IL-8 | IL-17 | IL-4 | IL-12 p70 | TNF-α |
| --- | --- | --- | --- | --- | --- | --- | --- | --- | --- | --- | --- | --- |
| CV-A10-0h | 0.84 | <0.83 | 0.3 | 1.54 | <1.69 | 0.56 | <0.79 | 2.17 | <1.27 | <0.46 | 0.48 | <0.77 |
| CV-A10-12h | 0 | 7.38 | 1.02 | **9.36** | **105.59** | 0.66 | 0.78 | 2.32 | 1.34 | 0.69 | 1.01 | 0.91 |
| CV-A10-24h | 0 | 5.58 | 0.83 | **11.66** | **126.29** | 0.43 | 0.84 | 5.52 | 0.69 | 0.59 | 0.45 | 0.4 |
| CV-A10-48h | 0 | 1.49 | 3.2 | **364.85** | **191.77** | 2.44 | 2.89 | **990.64** | 2.9 | 6.95 | 0.2 | 12.6 |
| CV-A10-72h | 0 | 3.28 | 3.2 | **403.48** | **216.98** | 2.43 | 0.71 | **1214.79** | 2.43 | 7.01 | 0.07 | 11.17 |
| CV-A10-96h | 0 | 5.3 | 4.69 | **566.02** | **322.46** | 2.52 | 0.43 | **2890.55** | 2.64 | 8.88 | 0.42 | 9.35 |
