# Supplementary material for: Coxsackievirus A10 blocks autophagosome-lysosome fusion to promote viral nonlytic spread and inflammatory cytokine release
Source: Microbiol Spectr. 2025 Oct 30;13(12):e00830-25. doi: 10.1128/spectrum.00830-25 (PMC12671134; doi:10.1128/spectrum.00830-25)
Supplement: Table S2 — Identification of inflammatory cytokines in CV-A10-infected HUVECs with different treatments. [file spectrum.00830-25-s0005.docx]

**Table S2. Identification of inflammatory cytokines in CV-A10-infected HUVECs with different treatments.**

| Groups | IL-5 | IFN-α | IL-2 | IL-6 | IL-1β | IL-10 | IFN-γ | IL-8 | IL-17 | IL-4 | IL-12 p70 | TNF-α |
| --- | --- | --- | --- | --- | --- | --- | --- | --- | --- | --- | --- | --- |
| CV-A10 | 0 | 5 | 4.64 | **584.07** | **279.09** | 2.46 | 1.9 | **2478.48** | 2.89 | 7.73 | 0.43 | 10.13 |
| 3MA+CV-A10 | 0 | 2.72 | 1.1 | **26.38** | **47.26** | 0.63 | 2.26 | 8.02 | 1.07 | 0.63 | 0.6 | 0.73 |
| GW4869+CV-A10 | 0 | 4.83 | 2.9 | **217.79** | **155.84** | 2.68 | 0 | **279.8** | 2.46 | 6.16 | 2.94 | 16.08 |
| CQ+CV-A10 | 0 | 3.52 | 3.16 | **213.84** | **156.83** | 2.7 | 0 | **252.89** | 2.2 | 6.19 | 3.17 | 16.95 |
| Bafilomycin+CV-A10 | 0 | 1.47 | 4.27 | **584.91** | **270.17** | 2.55 | 0 | **2154.2** | 2.49 | 8.1 | 0.43 | 10.75 |
