# Supplementary material for: Coxsackievirus A10 blocks autophagosome-lysosome fusion to promote viral nonlytic spread and inflammatory cytokine release
Source: Microbiol Spectr. 2025 Oct 30;13(12):e00830-25. doi: 10.1128/spectrum.00830-25 (PMC12671134; doi:10.1128/spectrum.00830-25)
Supplement: Table S3 — Identification of inflammatory cytokines in brain and lung tissues of mice. [file spectrum.00830-25-s0006.docx]

**Table S3. Identification of inflammatory cytokines in brain and lung tissues of mice.**

| Groups | IL-5 | IFN-α | IL-2 | IL-6 | IL-1β | IL-10 | IFN-γ | IL-8 | IL-17 | IL-4 | IL-12 p70 | TNF-α |
| --- | --- | --- | --- | --- | --- | --- | --- | --- | --- | --- | --- | --- |
| PBS-Brain | 0 | 0 | 0.95 | 2.52 | 0 | 0.39 | 2.69 | **21.88** | 0.56 | 0.59 | 0.93 | 0.4 |
| GW4869-Brain | 0 | 0 | 0.79 | 2.48 | 0 | 0.29 | 3.12 | **25.21** | 1.08 | 0.76 | 0.81 | 2 |
| CV-A10-Brain | 0 | **15.74** | **9.5** | **6624.95** | **418.21** | 2.95 | 1.41 | **6234.1** | 2.69 | **10.54** | **4.11** | **37.85** |
| CV-A10+GW4869-Brain | 0.6 | 5.32 | 4.87 | **3925.01** | **87.37** | 0.47 | 2.67 | **1926.55** | 0.84 | 0.78 | 1.16 | **34.46** |
| PBS-Lung | 1 | 1.62 | 1.59 | **27.4** | 4.22 | 1.19 | 2.68 | 2.1 | 5.93 | 1.23 | 1.73 | 2.05 |
| GW4869-Lung | 1.05 | 1.2 | 1.42 | **20.29** | 0 | 1.43 | 2.84 | 0.46 | 3.78 | 1.01 | 1.48 | 2.31 |
| CV-A10-Lung | 0 | **13.25** | 5.79 | **6690.02** | **338.64** | 2.82 | 0.48 | **4222.09** | 2.67 | **8.67** | 2.72 | **49.34** |
| CV-A10+GW4869-Lung | 0 | 2.66 | 4.55 | **463.58** | **238.43** | 2.54 | 0.61 | **1650.52** | 2.48 | 7.34 | 0.26 | 11.91 |
